# Teaching the Modeling of Human–Environment Systems: Acknowledging Complexity with an Agent-Based Model

**DOI:** 10.1007/s10956-022-10022-z

**Published:** 2023-01-16

**Authors:** Maria Haensel, Thomas M. Schmitt, Jakob Bogenreuther

**Affiliations:** University of Bayreuth, Professorship of Ecological Services, Bayreuth Center of Ecology and Environmental Research (BayCEER), Universitätsstraße 30, Bayreuth, 95447 Germany

**Keywords:** Higher education, University teaching, Blended learning, Individual-based models, Wicked problems, Socio-ecological systems

## Abstract

Agent-based modeling is a promising tool for familiarizing students with complex systems as well as programming skills. Human–environment systems, for instance, entail complex interdependencies that need to be considered when modeling these systems. This complexity is often neglected in teaching modeling approaches. For a heterogeneous group of master’s students at a German university, we pre-built an agent-based model. In class, this was used to teach modeling impacts of land use policies and markets on ecosystem services. As part of the course, the students had to perform small research projects with the model in groups of two. This study aims to evaluate how well students could deal with the complexity involved in the model based on their group work outcomes. Chosen indicators were, e.g., the appropriateness of their research goals, the suitability of the methods applied, and how well they acknowledged the limitations. Our study results revealed that teaching complex systems does not need to be done with too simplistic models. Most students, even with little background in modeling and programming, were able to deal with the complex model setup, conduct small research projects, and have a thoughtful discussion on the limitations involved. With adequate theoretical input during lectures, we recommend using models that do not hide the complexity of the systems but foster a realistic simplification of the interactions.

**Supplementary Information:**

The online version contains supplementary material available at 10.1007/s10956-022-10022-z.

## Introduction

Human–environment systems entail complexity regarding heterogeneity, interactions, and feedback mechanisms, creating wicked problems (Zellner & Campbell, 2015). Teaching the principles of complex systems can allow students to understand different scientific domains. Wicked problems have firstly been defined by Rittel and Webber (1973) as problems that cannot be finally formulated and lack a final solution, while intermediate solutions might generate new problems. They exist in human–environment systems because of differences in social ideals and uncertainties of environmental changes (Duckett et al., 2016). One approach to tackle wicked problems is via complex system modeling, for example, agent-based modeling. For these applications, the non-deterministic and iterative model-building process is an excellent fit (Zellner & Campbell, 2015). Multiple interdependencies of wicked problems can be represented in complex system modeling via the set of initial parameters, causalities, and produced outcome variables (Zellner & Campbell, 2015).

To familiarize students with such complex systems, agent-based modeling, an established tool in research, is also an evolving tool in education (Bodine et al., 2020; Ginovart, 2014). Complex agent-based models are less developed in human–environment sciences than in disciplines like chemistry, physics, or climate sciences (An et al., 2021). Therefore, education in this field is a worthwhile investment, potentially benefitting future research. Understanding complex systems will help students to understand not only human–environment systems but also other scientific domains, e.g., concepts related to self-organization (Rates et al., 2016).

Agent-based models (also called individual-based models) are characterized by individual actors (agents) making decisions and interacting with each other and their environment (An et al., 2021; Railsback & Grimm, 2012). They are carried out via a programmed code, but certain platforms with a graphical user interface allow the models to be run without understanding the underlying mathematics (Ginovart, 2014). Yet, complex elements like path dependency, self-organization, or feedback mechanisms can emerge that are not intuitively visible in the single parts of the system alone (An et al., 2021). Agent-based models are most commonly formulated in discrete events and time steps (Ginovart, 2014). They are suitable for education, as the outcome of theoretical concepts can be illustrated and changed in real time. For example, the open-source platform NetLogo can be used for running models without adapting the underlying code. At the same time, it is also suitable for teaching programming skills (Murphy et al., 2020).

Studies evaluating the usage of agent-based modeling in teaching have previously focused on simple models with a small set of adjustable parameters. This contrasts with understanding the capability of agent-based modeling to tackle wicked problems in classrooms. For teaching modeling in the social science domain, models on segregation are commonly assessed (Hostetler et al., 2018). In the ecological field, studies focus mainly on predator and prey models (Ameerbakhsh et al., 2016; Benhadi-Marín et al., 2020; Ginovart, 2014). Also, models for teaching basic principles of survival and population growth in terrestrial or aquatic ecosystems are evaluated (Dickes et al., 2016; Hmelo-Silver et al., 2014). The studies that evaluate teaching modeling practices are conducted with students ranging from elementary school (Dickes et al., 2016) to higher education (Ameerbakhsh et al., 2016). Students have described this way of teaching as interesting (Ginovart, 2014) and rated NetLogo as a game-based learning tool (Ameerbakhsh et al., 2016).

Complex human–environment models on the other hand are often used in research but are not evaluated for educational purposes. Agent-based models capturing this complexity mainly illustrate policies’ impact (Brady et al., 2012; Happe et al., 2011) and decision-making (Habib et al., 2016; Valbuena et al., 2010) on agricultural and forestry land use. To our knowledge, no assessment of whether students could deal with this complexity in the teaching agent-based models exists in the literature so far. Only Bodine et al. (2020) present a general framework for how complex agent-based models can be conveyed to students. Yet, an in-depth evaluation of students’ ability to deal with this complexity is lacking. Further research is therefore needed on the suitability of agent-based models in a classroom setting, with a larger set of adjustable parameters, displaying environmental as well as human interactions. This is of particular interest in higher education, in which students often have different levels of methodological capabilities and thematic knowledge.

This study aims to (i) present a complex model of a human–environment system developed for teaching in higher education (see "Method" section), (ii) to analyze the ability of a heterogeneous group of students to deal with the inherent complexity, and (iii) to discuss potential success factors of teaching complex systems.

## Methods

### Classroom Setting and Student Characteristics

For the exercise part of a course module on “Land use policies, markets, and ecosystems,” we—both the lecturers of the class and authors of the paper—pre-built a spatially explicit agent-based model named *World of Cows*. As an example of human–environment systems, we chose an agricultural landscape dominated by dairy farming. In this landscape, dairy farms influence and are affected by different ecosystem services (e.g., soil fertility and global climate regulation). As a governance tool, the model users can manipulate policy options—in this case, the students of our class. As learning objectives of the course, we defined that students can apply an agent-based land-use model based on real-world policies and markets and interpret its results in terms of impact on land use and ecosystem services. In addition, students should be able to reproduce the basics and fundamental concepts of modeling and put them in the context of various techniques.

**Fig. 1:**
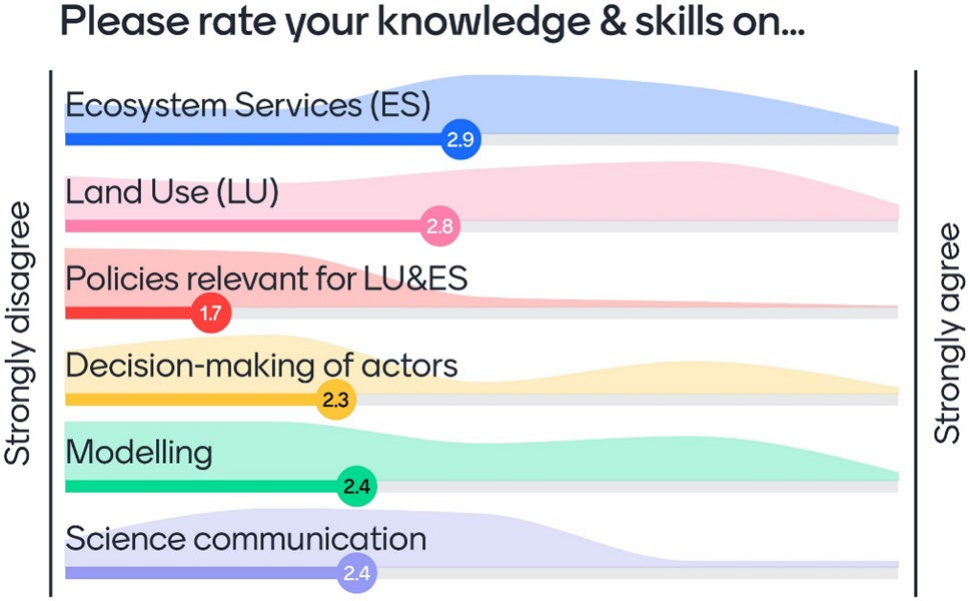
Results of a query shown to all students (*n* = 28) attending the lecture part of the module “Land use policies, markets, and ecosystems” (1 = strongly disagree to 5 = strongly agree), conducted with the interactive presentation tool Mentimeter (Mentimeter, 2021)

The course audience were international master’s students from the University of Bayreuth, Germany. Participating students (*n* = 18) had varying prior background knowledge on key topics of the course. They were enrolled in different master’s programs—both Master of Arts (International Economy and Governance) and Master of Sciences (Global Change Ecology, Food and Health Sciences, Environmental Geography, Environmental Sciences) and had completed bachelor’s degrees from a broad range of disciplines. In a query in the lecture part of the course module (Fig. 1), conducted with the interactive presentation tool Mentimeter (Mentimeter, 2021), the students could rate their background knowledge at the beginning of the course. The group faced strong heterogeneity regarding soft skills such as communication, hard skills such as modeling background, and thematic knowledge.

Considering this heterogeneity, e.g., in terms of modeling skills, the course started with general exercises illustrating principles of agent-based modeling. We then introduced the pre-built model in different steps, starting with a strongly simplified version. Using these versions, we conveyed the phases of model development, including finding coding errors and model calibration. The students learned to conduct experiments with the model (by iterating through different parameter settings) and how to run optimization procedures (to find optimal parameter settings). Toward the end of the course, we applied sensitivity analysis techniques and explored model validation strategies with the students.

### Agent-Based Model of a Human–Environment System: World of Cows

**Fig. 2:**
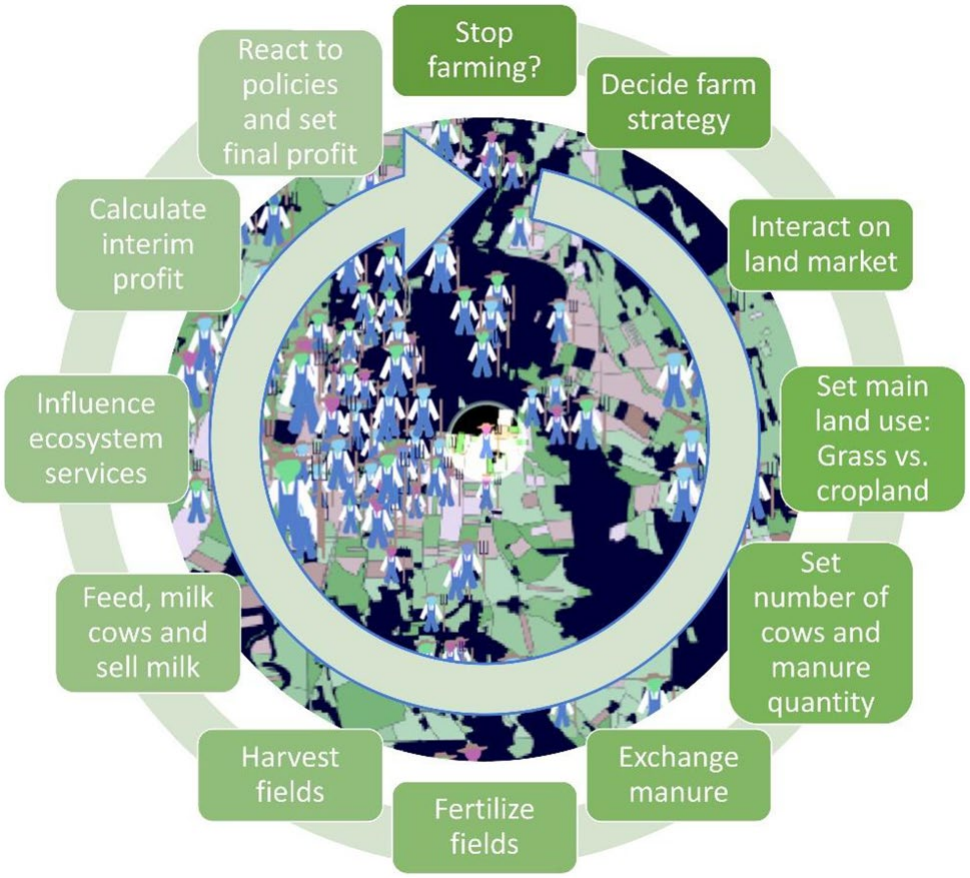
Different actions by the dairy farms in the model *World of Cows* within one year (equivalent to one time step in the model). Each year, the farms start by deciding if they want to stop farming. Each action is represented by one submodel. The background shows part of the “world” of the graphical user interface of the model in NetLogo (in the setup, based on real-world data for the agricultural fields)

**Fig. 3:**
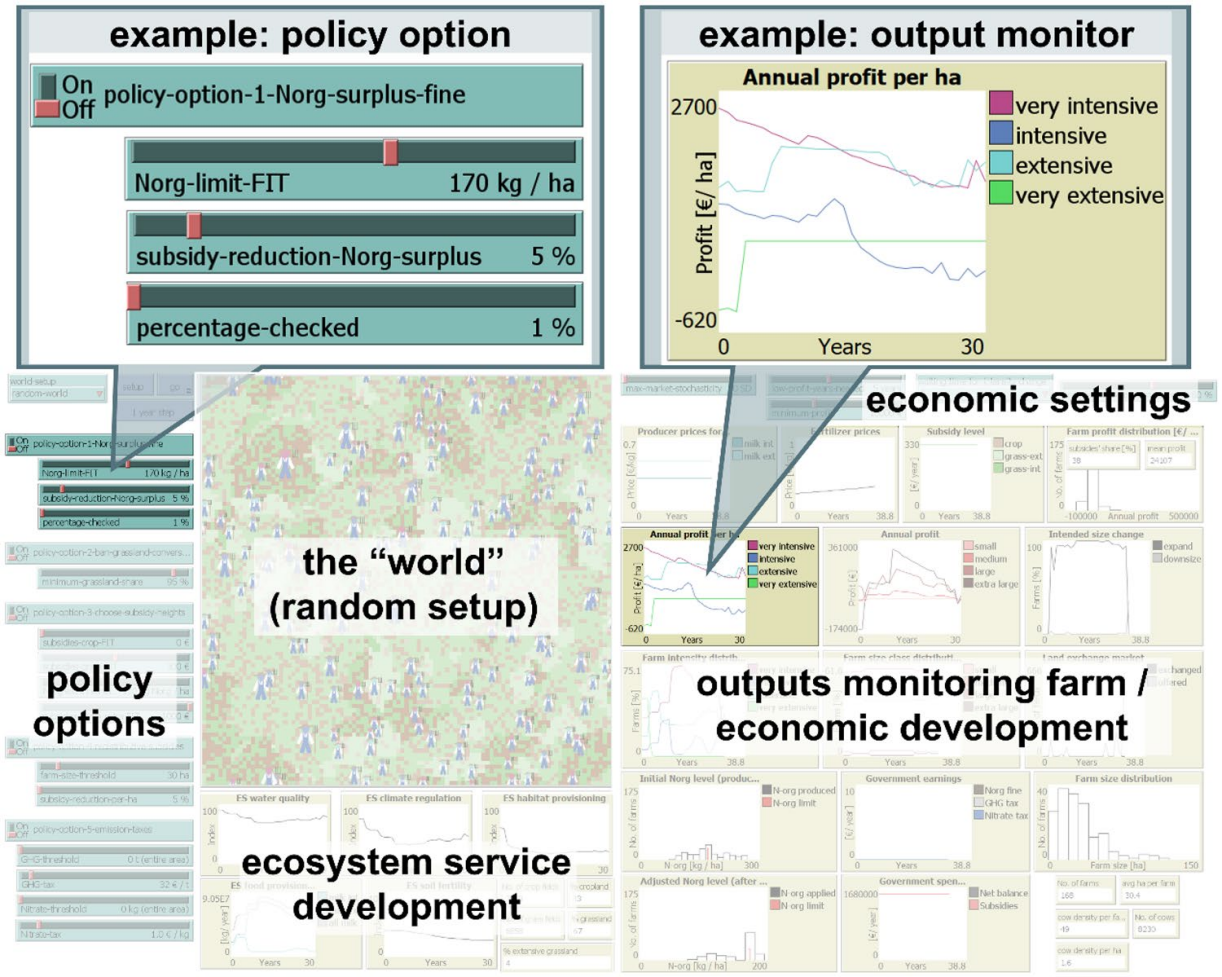
Sections of the model interface *World of Cows*. The users can adjust the policy options and economic settings through sliders and choosers. In the chosen example policy option, users can set a limit of organic nitrogen (Norg) per hectare, how much subsidies will be deducted if farms exceed it, and what percentage of farms are checked. Ecosystem services and farm indicators are presented in output plots and monitors. In the presented example output monitor, the profitability (in terms of Euro per hectare) for the different management intensity classes of farms is displayed. Here, the presented model “world” setup is based on the second alternative of a “random” configuration of agricultural fields (in contrast to Fig. 2)

The model’s principal purpose is to test the effect of different policy options on the provisioning of five different ecosystem services in an agricultural landscape dominated by dairy farming. The economic viability of dairy farms and overall government spending are also considered. The complexity of the model is shown in complex model behavior (e.g., non-linear relationships) and by a rather complicated model setup (Sun et al., 2016), which is expressed by a high number of parameters (pre-set: 43, chosen via interface: 5), state variables (dairy farms: 7; agricultural fields: 8, governance structure: 14), and submodels (12). The submodels are equivalent to the consecutive decisions and steps taken by dairy farms within one year (Fig. 2). The model can be operated in two “world” versions. One is based on anonymized real-world data with fixed shapes of fields and locations of farms (see background in Fig. 2), the other on a grid of fields with random placement of initial farms (see “world” segment in Fig. 3).

Farms can operate at four different intensity levels. These will, in turn, affect milk production and the level of regulating ecosystem services such as climate regulation. Additionally, there are two levels of milk producer prices depending on the intensity level, simulating organic and conventional farming. Based on income from two sources, farms decide if they stay in business: the net gain from selling milk plus government subsidies. Farms interact in a land tenure and a manure market. The policy options to influence the system are based on agricultural policies already implemented or discussed in a central European context.

**Fig. 4:**
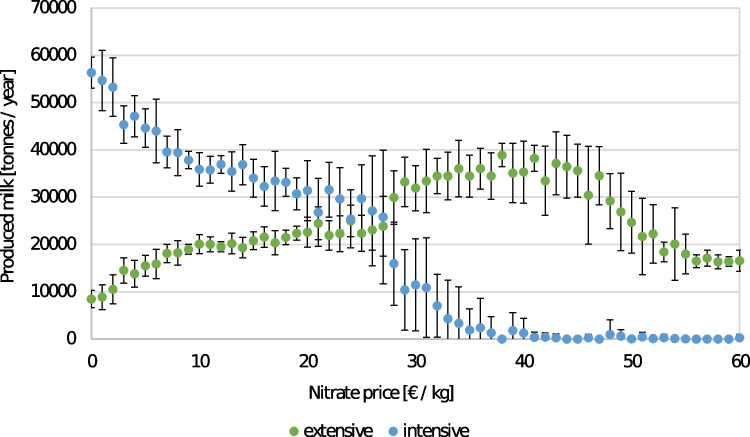
Effect of one of the five policy options (nitrate tax) in the model *World of Cows* on produced milk (originating from farms operating in different intensity levels)

One of the innovative policy options not yet implemented in a current real-world setting is a carbon or nitrate tax for agricultural production. This is one of the examples of how students could observe non-linear relationships between variables (Fig. 4). The model takes many processes and feedbacks into account. Still, we had to make many simplifications, e.g., about farmers’ decision-making, farm structures, and interaction effects.

The complete model description, including details on design concepts, submodels, and basic parametrization, is provided as an ODD + D Protocol (Müller et al., 2013) in supplement S1. The model itself is available in the CoMSES Model Library.^1^

### NetLogo—an Easy Access to Complex Modeling

The model was coded in NetLogo version 6.2.1 (Wilensky, 1999). The modeling platform, rated as one of the easiest to handle while still having relatively good computational modeling strength (Abar et al., 2017), comes with three tabs: the code, information about the model, and the interface. Buttons, sliders, and choosers on the interface allow the user to change the model parametrization without changing the actual code. Model outputs can be directly visualized in plots and monitors (Fig. 3).

Two convenient tools come with the modeling platform for more complex model analyses. With BehaviorSpace, experiments can be run with models in which the covered parameter space and the number of replications (in case stochastic elements are present in the model) can be chosen freely (NetLogo, 2021). BehaviorSearch,^2^ in turn, allows for optimization procedures. It can be used for model parametrization and analysis. In the context of our model, different “political goals” (e.g., the number of very extensive farms or a specific ecosystem service) can be optimized in terms of which policy settings are the best for achieving a certain goal.

### Group Work—An Indication of Students’ Capacity to Deal with Model Complexity

In group projects (teams of two), students were asked to set a research goal they could answer/accomplish with the pre-build model. In their group work, students could either focus on parametrizing the *World of Cows* to answer a specific research question (analysis-based approach) or on rewriting code in NetLogo by adding or modifying modules in the model script (code-based approach). In both cases, they were asked to use at least one of the two tools for their analysis: BehaviorSpace or BehaviorSearch. As a further prerequisite, impacts of policies on the provisioning of ecosystem services were to be analyzed with the model.

To explore how well students could deal with the model complexity, we analyzed the group work outcomes in the following steps: We checked (i) whether students set appropriate research goals for their group work, (ii) for matching content of their analysis or code adaptation with suitable methods, (iii) if plausible results were produced, (iv) whether model and group work limitations were properly discussed, and (v) if links to real-world challenges and policy suggestions were made.

## Results

In the following sections, the different student group work outcomes are evaluated as indicators of how well students could cope using a complex model to analyze the *World of Cows* as a representation of a human–environment system.

### Appropriateness of Research Goals

**Fig. 5:**
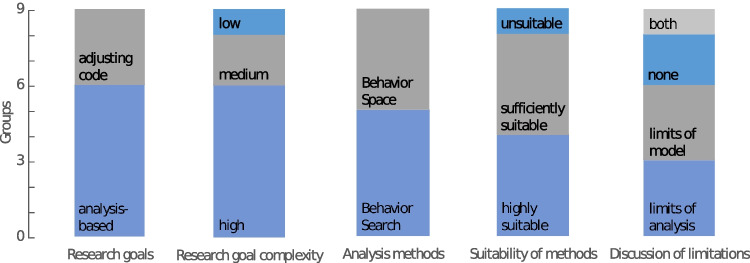
Counts of student groups showing the proportions of (i) approach and (ii) complexity of research goals, (iii) tools used for the method of analysis, and (iv) their suitability to reach the defined research goal, as well as (v) discussed types of limitations

We rated all research goals set by students in their group work as appropriate for the model analysis or adjustment of model components. To our understanding, they all showed that students understood the tasks and possibilities of applying an agent-based model of a human–environment system. The research goals covered a wide range of topics (see Table 1) and differed in the scope of model adjustment and depth of analysis (Fig. 5), but all fulfilled the baseline criteria. Some student groups investigated the provisioning of multiple ecosystem services (*n* = 4) under policy scenarios or after code adjustments. Others focused on specific ecosystem services, namely soil fertility (*n* = 3), habitat provisioning (*n* = 1), or climate regulation (*n* = 1). The approaches considered either multiple policy options (*n* = 5) or a single policy mechanism (*n* = 2). Three groups aimed at improving the model itself by (i) adding a social norm of intrinsically motivated extensive farming, (ii) optimizing the soil quality factor in the model, or (iii) including cow mortality due to heat waves. With these code-based research goals of improving the model, the respective student groups showed a deeper understanding of the modeling work than the other groups that decided on the analysis-based research goal. We rated the complexity of set research goals generally as high (*n* = 6), based on the number of policy options and ecosystem services considered and if trade-offs of policy effects were included and the code was adjusted. According to our assessment, the research goals of some groups only showed a low (*n* = 1) or medium (*n* = 2) complexity.

**Table 1:** Outcomes of the final project of student teams

**Research goals**	**Methods**	**Results**	**Discussion of limitations**	**Real-world applicability**
Best policy setting for regulating ecosystem services?	Optimization of joint ecosystem services-index per policy option (BehaviorSearch).	Best performing policy option is a GHG and nitrate tax, followed by subsidies for extensive grassland. The optimal policy settings led to low food provisioning services.	Random model components & methodological approach. Missing economic consideration.	Food provisioning is essential in real-world agricultural systems. Therefore optimal solutions should not be directly translated into policies.
Best policy setting to maximize habitat provisioning and viability of cow farms?	Optimization for three individual and combined policy options (BehaviorSearch).	Grassland conversion ban is the single best policy option but can be improved when combined with subsidies.	Implicitly via impact of policy settings.	Proposition that there should be one targeted policy rather than a policy mix to avoid too strong impacts on profitability.
GHG tax rate for optimal climate regulation?^a^	Optimization of climate regulation (BehaviorSearch).	Best results can be obtained at a GHG tax rate of 251 €/t.	Potentially missed global maximum (because of chosen steps of 25 €/t for GHG tax rate).	Comparison to suggested (drastically lower) CO_2_ price range by the German government & mentions exemption of agriculture.
GHG tax rate leading to a significant change in ecosystem services?^a^	Ecosystem services per GHG tax rate level (BehaviorSpace) Double-sided *t-*tests.	Threshold of significant effects differ: For regulating ecosystem services between 100–120 €/t, milk production already significantly decreases at 70 €/t.	–	GHG prices were based on real-world examples (e.g., Germany, Switzerland, Sweden). Conclusion that current German CO_2_ price of 25 €/t is too low.
How to maximize soil fertility through policy measures?	BehaviorSpace: Baseline of soil fertility index (BehaviorSpace) & Optimization of soil fertility per policy option (BehaviorSearch).	Banning grassland conversion leads to a higher soil fertility than (high) subsidy payments.	–	–
How to maximize soil fertility by regulating policies that affect organic nitrogen?	Optimization of soil fertility per policy option (BehaviorSearch).	A 30% increase in soil fertility (compared to almost no change in the baseline) could be reached with a combination of a regulatory policy, a threshold of subsidy support for extensive management, and a nitrate tax.	Implicitly via model logic of how organic nitrogen processes are implemented in the model.	Regulating policy showed the best effect, but only if many farms were checked (might be challenging to implement). Link results to government report.
**Adjust code**: Improved soil-fertility-factor distribution based on real-world data	Performance of new soil-fertility-factor (BehaviorSpace).	Using real-world data, the soil quality rating, the milk output, and profits per hectare are higher.	Soil data would be needed in higher resolution.	–
**Adjust code**: Influence of heatwaves on mortality of cows	Effects of heatwaves on cows (BehaviorSpace).	Number of cows is decimated in years with heatwaves. Impact on income is moderated by subsidies.	No feedback of cow-mortality on farmers’ decision-making.	–
**Adjust code**: Social norm of intrinsic motivation for extensive farming. Which policies support extensive farms?	Farm intensity with different policy settings (BehaviorSpace).	With the new module on intrinsic motivation, (very) extensive farms were best supported with a policy mix including regulations and subsidies.	Other factors might influence farmers’ decision-making (than included in the model).	There are cases of organic farmers switching back to conventional farming, so persistence (due to intrinsic motivation) is not always the case.

^a^GHG (greenhouse gas) tax rate is indicated in Euro (€) per ton (t) of CO_2_-equivalents

### Suitability of Methods

According to our evaluation, the largest part of the student groups chose sufficiently suitable (*n* = 4) to highly suitable (*n* = 4) methods, and the content of the analysis or code adaptation matched the research goals well. All student teams used at least one of the offered analysis tools (BehaviorSpace and BehaviorSearch), depending on their specific research interests (see Table 1). One team had major difficulties during the course in understanding the usage of the tools and did not perform them sufficiently to pass the course in the initial attempt. After thorough feedback from the lecturers, the analysis for the second attempt was more profound, and BehaviorSpace was used in a correct way. The three teams with a code-based approach adjusted the pre-written NetLogo code. One team inserted conditional statements to give farmers the option to manage their grasslands extensively. This way, they allowed for intrinsic motivation for “environmentally friendly farming,” even if it is not profitable. Another group added an improved soil fertility factor of the fields for the study as an alternative to a random assignment of soil fertility per field. A third group introduced cow mortality due to heat waves in the model.

**Fig. 6:**
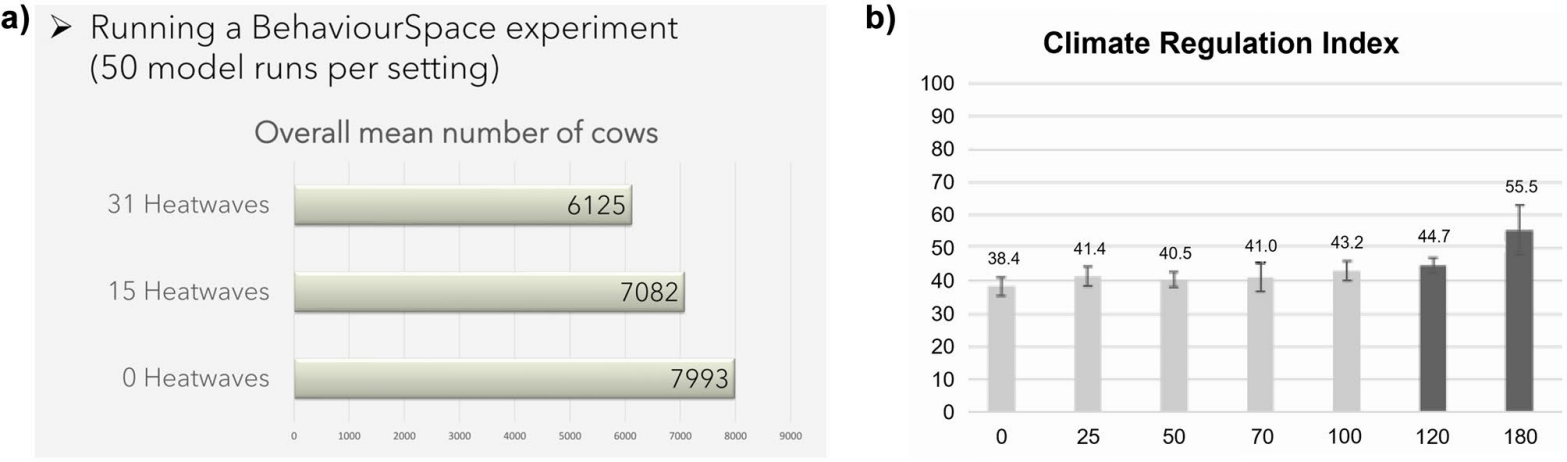
Two examples of group work results showing **a** an identification of the impact of cow numbers through heatwaves and **b** the height of a greenhouse gas tax (Euro per ton CO_2_ equivalents) needed to significantly impact ecosystem services. In this case, climate regulation (120 €/t)

Most group work results (*n* = 8) were presented with descriptive statistics obtained in BehaviorSpace and/or BehaviorSearch. For example, one group compared mean number of cows depending on the occurrences of heatwaves (Fig. 6a). Only one group conducted a more profound statistical analysis. They used standard deviation, and a double-sided t-test to assess the impacts of greenhouse gas tax rates on the provisioning of ecosystem services (Fig. 6b). No teams conducted a sensitivity analysis of their results.

### Plausibility of Results

All group work results (see Table 1) were rated by us as generally plausible outputs, given the framework of the pre-written model. However, for more than half of the groups (*n* = 5), we felt unsure if all steps in the analysis were carried out correctly because of different potential sources of errors like (i) a low number of replicates even though a strong stochastic fluctuation of marked prices was set, (ii) poorly specified ranges of modeling parameters, or (iii) impression of unclear understanding of some of the model components.

### Acknowledgment of Limitations

Most student groups (*n* = 7) pointed out at least one limitation, such as simplified assumptions used in their analyses and methodological procedures (see Table 1). Some groups set their focus on limitations of their own approach, like (i) chosen interval of values in the optimization procedure, (ii) low resolution of available data, and (iii) one-sided focus of the analysis. Some groups rather pointed out limitations of the pre-written model *World of Cows* as such, like (i) limited complexity of farmer’s decision-making, (ii) simplified representation of geo-chemical processes, and (iii) random model components like implemented fluctuating market prices that make the analysis and prediction more challenging.

### Links to Real-World Challenges and Policy Suggestions

A link to real-world challenges (see Table 1) was made by the majority of student groups (*n* = 6). Concrete policy suggestions were given by four groups, with one group also indicating possible limitations to a successful implementation. Another group did not want to base any policy recommendation on their model analysis because they felt it was too one-sided as they had left out economic considerations. One group also mentioned that in the near future, it would be necessary to update their analysis because of the changing legal framework of the common agricultural policy of the European Union in the next funding phase. As part of the discussion of limitations (see chapter 3.4), also a comparison with real-world data, like government reports or statistical data, was made. Two out of the three groups not pointing out any real-world challenges had chosen a code-based approach. Real-world applicability was a less important question for these groups because of the technical focus.

## Discussion

### Most Students Successfully Handled Model Complexity

The outcomes of student group work indicate that teaching modeling of human–environment systems with a complex agent-based model setup has challenges but is well achievable. Within one semester, even students with little background in modeling and programming could deal with the complicated model structure and complex model behavior (Sun et al., 2016). Most students provided thoughtful discussions as part of their small research projects. These included comprehensive analyses of policy scenarios on land use and ecosystem services as well as adding new model components. Turner et al. (2021) have highlighted the need for interdisciplinary teaching approaches for complex problem-solving. Their experiences with a multi-university cohort (using system dynamics modeling) support our study results that teaching complex systems does not need to rely only on simplistic models.

Our chosen indicators of how well the students could deal with model complexity all showed satisfactory levels. Especially high indicator values were reached for (i) choosing appropriate research goals, (ii) the principal suitability of applied methods, and (iii) the acknowledgment of limitations of the analysis. Students used most, but not all, methodologies introduced in class. That no group work included a sensitivity analysis might be due to the late introduction of this method, but it was likely also perceived as a challenging additional effort. We rate the indicator of acknowledging limitations as particularly important in showing if students understood model complexity. Holmes et al. (2015) also used this as an indicator of student’s ability to think critically. Two groups in our study did not point out any limitations in their group work presentation. This could indicate a lack of in-depth understanding of the model complexity by the respective groups. Hogan and Thomas (2001) rated (high school) student groups that failed to investigate properly why a model yielded a certain output as a “less productive” modeling approach. A sound model interpretation and being able to identify improvement needs are therefore closely linked. Most of the student groups in our study proved, by pointing out at least one and often several limitations of their results, knowledge about the model structure and insights into relevant impact pathways of the chosen policy options.

We perceived that correctly using the two built-in analysis tools (BehaviorSearch and BehaviorSpace) in NetLogo was the most challenging part of the course work for the student teams. Murphy et al. (2020) mention the step of designing experiments in BehaviorSpace as one of the more advanced elements of a teaching schedule using agent-based models, requiring substantial previous understanding and exercise by students. In our experience, using the optimization tool BehaviorSearch (not included in the analysis by Murphy et al. (2020)) was even more challenging for students. Students’ struggles with the two analysis tools negatively affected our rating of the plausibility of group work results in terms of how reliable we perceived the results. However, in our eyes, the principal importance was that students correctly understood and handled the policy options and ecosystem service indices represented in the model. This was the case for the large majority of student groups. According to our understanding, establishing connections from the model results to real-world applications hints toward a deeper understanding of the motivation behind modeling human–environment systems and its implications. Gill et al. (2014) also used this as an indicator of depths of understanding. In their case, they analyzed how well 7^th^-grade students could apply knowledge to real-world problems and decision-making. In our study, such a link was made by more than half of the student groups. We rate this as a convincing performance.

### Success Factors for Teaching Complex Systems to a Heterogeneous Group of Students

We identified several factors that contributed to successfully teaching agent-based modeling to students without compromising on the complexity involved. These are also based on students’ course evaluations and individual feedback at the end of the course. The most critical factors were offering a pre-built model, choosing an accessible coding environment, using flipped-classroom elements, and flexible group work projects.

For our class, we provided students with a pre-built model that the students could engage with and modify. Mulder et al. (2015) identified that providing high-school students with an outlined model they needed to complete enhanced their learning experience in contrast to starting the modeling from scratch. Offering a pre-built model was key to teaching the complexity of studying human–environment systems in the class. As several students had little to no prior programming skills, providing a model in various stages throughout the semester helped them to follow the modeling process. This would not have been possible if they had to write the model from scratch. Alessi (2000) defined this approach of students using a pre-built model as suitable for procedural learning while building a model by themselves rather suitable for declarative or conceptual learning. Before students focus on complex coding questions, we perceive it as very helpful for them to understand all aspects of modeling practice defined by Schwarz et al. (2009), namely constructing, using, evaluating, and revising models. We covered all of these aspects in our course, especially the three later ones. A possible follow-up course, teaching students how to build a complex model, can therefore integrate what the students learned in our class but would need an additional introductory course diving deeper into coding languages. We used the comparably simple coding language NetLogo, which was a great advantage for students to get familiar with agent-based modeling compared to other, more difficult coding environments (Abar et al., 2017; Railsback & Grimm, 2012). These considerations made complex agent-based modeling accessible to a group of students with very heterogeneous backgrounds. As we assumed only little prior capabilities in modeling, statistics, or thematic knowledge, our approach should also be feasible to apply in other contexts, including undergraduate education. However, as skills in independent working and knowledge of human–environment systems are necessary to understand the model, some adjustments might be required if used in undergraduate or even secondary education.

One of the major challenges faced in the course was the very heterogeneous group of students with various disciplinary backgrounds and study progress. To deal with this heterogeneity, using group work proved to be one of the key factors. This relates to the results of Bodine et al. (2020), who found that even if students do not finish the task of completing a model, working on the topic in a group enhances the learning process. Giving students the flexibility to design their own research questions and to focus either on a methodological or theoretical task also seemed to be a very suitable way of addressing the heterogeneity in the class without compromising students’ learning success. Due to the heterogeneous group of students, we explicitly decided to teach large parts of the module in a flipped-classroom format, which proved to provide a very suitable learning environment. In a flipped classroom approach, much of the teaching material is provided to the students as preparatory material that needs to be studied before the class sessions, which can then be successfully used for more interactive learning in small groups (Wipper, 2021). The format has been gaining widespread attention in different higher education disciplines recently and is very suitable for teaching complex content. For example, Mattis (2015) found that teaching mathematical complexity to university students in a flipped classroom format increased accuracy and decreased mental effort in students’ work. In our study’s flipped-classroom sessions, students could learn the material at their own pace, skip basics, or consult additional material according to their previous knowledge. Students stated that they dedicated five to six hours on average for coursework, including time spent in class, with one person only spending less than three hours and one person nine to ten hours per week. The course was conducted during the first winter term of the Covid-19 pandemic. While the first few lessons could be conducted in person, we taught most of the course online.

### Methodological Limitations

In this study, we used our classroom as a practical case study to investigate the suitability of teaching complex human–environment systems in higher education. We employed a less common methodology by analyzing students’ group work outputs without interviews or questionnaires. To our understanding, this proved to be a very suitable approach. Nevertheless, we acknowledge that this offered no option for analyzing a controlled treatment effect. Additionally, limited understanding by single students might not be evident from group work results if they teamed up with a more advanced group partner. In future studies, we suggest adding student reflections, e.g., by including autoethnographic reports by students (Murphy et al., 2022). To achieve comparability of students’ capacity to deal with complexity, we only analyzed the results of one student cohort. Considerations were changing course and module requirements between terms and potential differences between classes with in-person and online teaching necessary due to the Covid-19 pandemic. The focus on one student cohort limited the number of students to only 18 students, which is a relatively small sample size. We recommend further research investigating how different cohorts of students can deal with complex models using agent-based modeling and whether these results are similar to our findings. Lastly, we recognize limitations related to online teaching due to the Covid-19 pandemic. Although we used activating elements and break-out rooms, online teaching might have limited the interaction between students and teachers. We expect that this might have slightly reduced the ability of students to deal with the complexity of the model.

## Conclusion

Teaching the principles of complexity can help students to understand different scientific domains and learn how to deal with today’s wicked problems like climate change. We presented the neglected approach to teach this real-world complexity with a correspondingly complex agent-based model (in terms of adjustable parameters and interdependencies). Our results show that we can trust students’ ability to deal with model complexity, even in an interdisciplinary course. This should encourage more teaching and research endeavors to take this route. We found that agent-based modeling is a suitable method for conveying complexity and wicked problems. According to our understanding, it can also be a helpful approach in other scientific domains and educational programs beyond the analysis of human–environment systems. Our chosen teaching approach proved to work well, especially for a heterogeneous group of students. We allowed students to develop their own research goals and introduced complex models stepwise with the flipped classroom technique. Our identified success factors can be applied to teaching modeling approaches in general, even though we perceive agent-based modeling to be a particularly useful tool.

## Supplementary Information

Below is the link to the electronic supplementary material.

Supplementary file1 (PDF 515 KB)

## Data Availability

The analyzed student group work results cannot be shared publicly. However, the complete code of the agent-based model used in this course (“World of Cows”) is available in the CoMSES Model Library https://www.comses.net/codebases/b67c5833-96fb-4c11-a37b-9034b7d58236/releases/1.0.0/.

## Footnotes

1 https://www.comses.net/codebases

2 See website for further information: https://behaviorsearch.org/
