# Supplementary material for: Teaching the Modeling of Human–Environment Systems: Acknowledging Complexity with an Agent-Based Model
Source: J Sci Educ Technol. 2023 Jan 16;32(2):256–66. doi: 10.1007/s10956-022-10022-z (PMC9842197; doi:10.1007/s10956-022-10022-z)
Supplement: Supplementary file 1 — Supplementary file1 (PDF 515 KB) [file 10956_2022_10022_MOESM1_ESM.pdf]

Supplementary Information:

ODD + D protocol of model description according to Müller et al. (2013)[1]

Teaching the modeling of human-environment systems: Acknowledging complexity with an agent-based model

Published in the Journal of Science Education and Technology

(2023)

Maria Haensel, Thomas M. Schmitt and Jakob Bogenreuther

Authors affiliation:

University of Bayreuth, Professorship of Ecological Services, Bayreuth Center of Ecology and Environmental Research (BayCEER), Universitätsstraße 30, 95447 Bayreuth, Germany

[1] Müller, B., F. Bohn, G. Dreßler, J. Groeneveld, C. Klassert, R. Martin, M. Schlüter, J. Schulze, H. Weise, und N. Schwarz. (2013) Describing human decisions in agent-based models–ODD+ D, an extension of the ODD protocol. *Environmental Modelling & Software* 48: 37–48. https://doi.org/10.1016/j.envsoft.2013.06.003

ODD + D Protocol for the Agent-Based Model *World of Cows*

I. OVERVIEW

I.i Purpose:

The purpose of the model is to analyze the influence of policies and markets on land use decisions of dairy farms. The land use decisions made, determine the delivered ecosystem services on the landscape level. The user can choose a combination of five policy options, as well as how strongly market prices fluctuate. A suitable choice of policy options simultaneously fulfills the following three “political goals” i) economic viability of dairy farming, ii) safeguarding ecosystem services at a certain level, and iii) spending as little money as possible on subsidies. The model has been designed for students to practice agent-based modeling and its respective output analysis.

I.ii. Entities, state variables, and scales

The model comprises a human-environmental landscape consisting of three types of entities: dairy farm agents (representing farming households), field agents (representing land parcels), and a governance structure (observer setting exogenous factors).

Dairy farms’ state variables include the farm location, a farm id (linking the farm to its fields), a set of fields that belong to the farm, the number of cows that the farm owns, received (traded) manure, its intended farm size change (downsize, expand or no change) and an intensity scale (from 1 to 4). The fields’ state variables are the location, the size (in ha), a field id, the id of the farm that the field belongs to (tenant), a factor of soil fertility, its land use (grassland vs. cropland), the amount of organic and mineral fertilizers used on the field (in kg N/year), as well as the number of cuts the field receives (if grassland). The governance structure is represented by five different policy options, including i) a fine on organic nitrogen surplus, ii) a ban on grassland conversion, iii) subsidy heights for different land uses, iv) redistributive subsidies, and v) emission taxes. Each of these also encompasses several options regarding the exact settings for the respective policy option (e.g., level of fine for organic nitrogen surplus).

The model is a spatially explicit model, taking into account the exact location of farms and fields. The user can choose between a real-world example and a random world setup (random distribution of farms and allocation of fields around farms). Each time step corresponds to one year. One model run covers a period of 30 years.

I.iii. Process overview and scheduling

Within each year, a sequence of activities takes place in the following order (see Fig. 1): First, each dairy farm decides to continue or to stop farming. The next step is a decision on the farm’s strategy in terms of changing either its intensity or size. This decision is based on its annual profit in relation to the profit of the majority of other farms. Based on the farm strategy, the next step is an interaction on the land market trying to either lease new or give away land, followed by updating its farm size. Consequently, the farm decides on the land use of its fields (depending on the chosen farm intensity), whether they are cultivated with crops or utilized as grassland. Depending on the new farm size and intensity scale, the farm specifies the number of cows it will hold in the upcoming year and how much manure its cows produce.

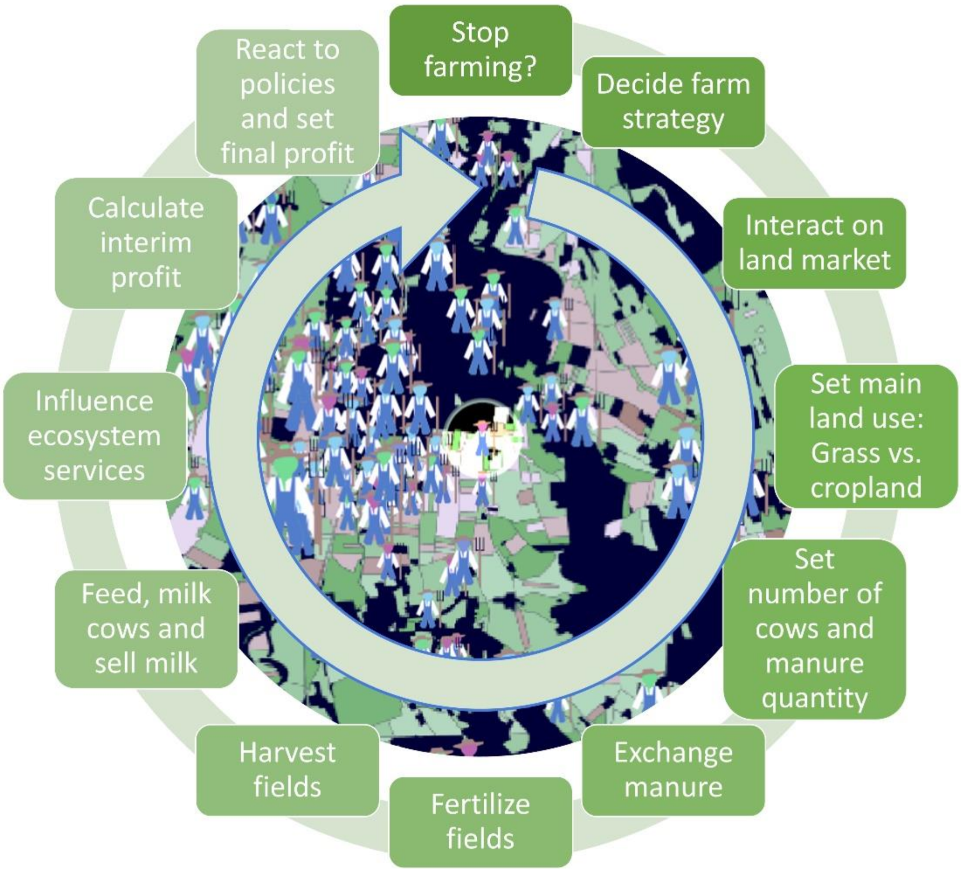

Fig. 1 *Different actions by the dairy farms within one year (equivalent to one time step in the model). Each year, the farms start by deciding if they want to stop farming.*

The farm will then interact on the manure market and potentially exchange manure with other farms. Following this, the farm fertilizes its fields with organic and mineral fertilizers, after which farms harvest their fields, determine the feed value of the grass, feed the cows, and determine the volume of milk their cows produce. The milk will then be sold at a set market price generating income for the farms. Before the farm's profit is calculated (depending on market prices and policy settings), the impacts of the land use decisions on a selected set of ecosystem services are determined.

II. DESIGN CONCEPTS

II.i Theoretical and empirical background

The underlying background of this agent-based model is to function as a tool for the impact assessment of policy and market instruments on land use and a selected set of ecosystem services. The modules on decision-making and interactions on land and manure markets are restricted by the limited information available to dairy farms. They have no foresight into the future and are satisfied when reaching a certain minimum income, which can be assessed in relation to their peers. The observer's policy and market decisions are linked to real-world cases but are still greatly simplified. It is furthermore assumed that a central authority owns all land, and farms do not own but only lease land.

With the decision style "based on profit", a special form of profit maximization was used as a behavioral theory. Farmers' decision-making for the two strategy decisions – size and intensity – impact all the following steps in each time step. How the profit maximization is implemented is based on the average profit of the other farms. Per size class (four classes)

as well as per intensity scale (four levels of intensity), the financially most successful strategy is determined. If the annual income of a farm is below a certain percentage (the percentage can be set by the user to a value between 10% and 90%) of the most successful strategy, it changes – under certain conditions[2] – its strategy in the direction of the most successful strategy.

This decision style was chosen due to its simplicity but also due to its similarity to real-world decisions. Imitation of other, more profitable farm strategies is widely seen as a prominent decision style (Le et al. 2010). The level of decision-making is on the farm scale.

II.ii. Individual decision-making

The subjects of the model are the farms deciding on the state of the fields. The observer makes further decisions in terms of policies and markets that affect the decision-making of the subjects as well as the state of the fields. The farms do not have any further, specific objectives to follow besides reaching a certain annual profit in relation to their social environment. The farms adapt their behavior depending on the decision style and decision strategy taken, but also depending on the situation of the land and manure market as well as depending on given policies and market scenarios.

Social norms influence behavior through the responsibility to follow policies. Cultural values are not further reflected in the decision-making. Space is explicitly included in the decision-making as the distance between the farm and the fields plays an essential role in deciding on fields to lease and its overall income. Temporal aspects are essential for decision-making, depending on whether the observer allows intensity changes every year, every three or five years. Also, the decision to stop farming is based on a number of years (set by the observer) and hence requires memory of the farms. Uncertainty is not explicitly included in the decision rules. Nevertheless, farms base their decisions on the income distribution of farms in the previous year. Hence, they take a certain risk as they do not know about the current year's land market, manure market, and price developments.

II.iii Learning

No individual or collective learning is included in the model

II.iv. Individual sensing

Dairy farms sense the annual profit of other farms as described under individual decision-making. Additionally, farms sense the productivity of land, the distance to fields, the policies that are in force, the market value of milk, prices for land as well as manure. Information are updated every year.

II.v. Individual prediction

No individual prediction is included in the model

II.vi. Interaction

Interaction between dairy farms takes place in the land and manure markets. The interaction depends on spatial distance, the amount of resources available, and the policies set by the observer.

II.vii. collectives

No collectives are included in the model.

---

[2] The intensity change of dairy farms with less than five cows is automatically set to intensify one step. In addition, the observer can choose via the interface to allow farms either to change their intensity scale every year or every five years (stressing that e.g. the conversion from conventional to organic farming is a longer-term process).

II.viii. Heterogeneity

Dairy farms have different goals (based on their individual decision-making) and act differently depending on the farming style (intensity scale) they follow for the year. Fields have different outputs depending on their productivity and their land use (cropland versus grassland).

II.ix. Stochasticity

In the random world setup, the farm location, the livestock density, and the allocation of fields to nearby farms are determined in a stochastic process. In addition, the market prices (producer prices for milk and fertilizer prices) can be chosen to fluctuate randomly.

II.x. Observation

The development of land use, amount, and size of farms can be observed at the GUI. Furthermore, the observer can follow the development of milk, and fertilizer prices, subsidy levels for cropland and grasslands, farm and profit distributions, government expenditures and earnings, as well as impacts on ecosystem services.

III. DETAILS

III.i. Implementation details

The model was written and implemented in NetLogo 6.0.4 , including a GIS extension.

III.ii. Initialization

The model can be initialized in two different versions, which the observer can decide upon before setting up the world. In the "random-world", 175 farms (with random farm sizes), approx. 5000-6000 cows and 10101 fields are distributed randomly in the world. The farms are categorized into 4 size classes (small (< 20 ha), medium (> 20 ha and < 75 ha), large (> 75 ha and < 100 ha), extralarge (> 100 ha)). If at least 3 large farms were created, neighboring fields are assigned to 0 to 3 of them until they are extralarge with a size between 100 ha and 125 ha. The grassland share of each farm is assigned approximately (with a normal distribution) in accordance with its intensity scale (see Table 1). In the "GIS-world", 145 farms, 4949 cows, and 923 fields are distributed realistically. The "GIS-world" is the same at any setup and not subject to change, while the distribution of the "random-world" is subject to randomization. The initial farm and cow density per hectare of the two worlds is approximately similar and hence comparable to a certain degree. The initial state of ecosystem services is set to an index of 100.

Farms are initialized into four different categories. In the "GIS-world", 41 farms are very-extensive, 42 are extensive, 13 are intensive, and 48 are very intensive. In the random world, the distribution varies. The intensity scales are defined in Table 1.

III.iii. Input data

The model uses GIS data as input data for the model setup in the GIS version (study region in Southern Bavaria). Several values used in the model needed parametrization. A complete overview of these values, including information on which submodel they were involved in as well as how and from which source they were obtained, can be found in Table 2 at the end of this protocol.

Table 1 *Definition of farm intensity scales and its respective attributes*

Scale	*Intensity*	*Cows/ha*	*Grassland share*	*Mineral fertilization?*	*Number of cuts (grassland)*
**1**	Very extensive	0.2 - 0.79	100%	No	1
**2**	Extensive	0.8 - 1.39	~ 85%	No	2
**3**	Intensive	1.4 - 1.69	~ 70%	Up to 200 kg N/ha	3 - 4
**4**	Very intensive	1.7 - 2.5	~ 50%	Up to 300 kg N/ha	4 - 5

III.iv. Submodels

III.iv.i Stop farming?

At the beginning of each year, farms decide whether they must stop farming. A farm stops operating after a certain amount of years in a row with a low profit. The number of years needed (1-10 years), as well as the minimum profit (5000-20000€), can be set by the observer at the interface.

III.iv.ii Decide farm strategy

Dairy farms must determine whether to continue with their current intensity and size or adjust these. For this decision, which is based on profit, each farm compares its annual profit to the profit of other farms. For the four size classes (small, medium, large, extralarge) and intensity levels (see Table 1), the mean annual profit is given as a global variable. If the farm's annual profit is below a certain threshold (to be set by the observer via the interface) of a different class's income, it will change its strategy one step in the direction of the most successful farm class. For the farm's intensity, however, the observer can choose via the interface whether the farm is allowed to change its intensity scale every year or only every five years. A farm size change is possible every year by default. Finally, there is one special case for intensity changes: Farms with less than five cows have to intensify.

III.iv.iii Interact in the land market

If the farm's intention is to change the farm size, the farm will interact in the land market. With an intention to decrease farm size, 10% of the most distant fields (number of fields, not the number of hectares) will be labeled "for-lease". If the farm only has five or less fields left, one field (the most distant one) will be set "for-lease". Fields of farms that stopped farming are now also labeled "for-lease". Fields are then distributed to farms (by annual income in descending order) that have the intention to increase their farm size until either all tenants are satisfied, or all fields in the list are taken. This is because successful farms with a high income are also more powerful and more engaged in the land market. The farms always choose the fields closest to the location of the farm.

III.iv.iv Set main land use

Depending on whether a grassland-conversion-ban is enabled (see submodel in policy implementation below), the farm decides every year whether fields are cultivated with crops or as grassland. With an enabled grassland-conversion ban (only conversion from cropland to grassland possible):

- very-extensive farms only cultivate grasslands.
- extensive farms make sure that at least 85% of their fields are grasslands.
- intensive farms make sure that at least 70% of their fields are grasslands.

- very intensive farms make sure that at least 50% of their fields are grasslands.

With no grassland-conversion ban enforced (conversion from cropland to grassland as well as from grassland to cropland is possible):

- very extensive farms only cultivate grasslands.
- extensive farms make sure their grassland share is equivalent to ~ 85% of their fields.
- intensive farms make sure their grassland share is equivalent to ~ 70% of their fields.
- very intensive farms make sure their grassland share is equivalent to ~ 50% of their fields.

III.iv.v Set number of cows and manure quantity

Dairy farms set the number of cows that the farm holds. This is based both on the previous decision on how much land the farm is leasing as well as the intensity scale in terms of how many cows per hectare the farm owns. Cows are allocated to farms randomly within the ranges defined in Table 1.

III.iv.vi Exchange manure

Dairy farms interact in the manure market. This is based on the balance of organic nitrogen of the farm. Firstly, the manure pool is created based on the excess manure of the farms which produce more manure than could legally be applied to their fields (the default limit is 170 kg N/ha, but can be adjusted via the interface if policy option 1 is active). Secondly, a list of interested 'receivers' is created for farms that are below the limit and would like to fertilize their fields with more manure. This list contains only intensive and very intensive farms, as they are assumed to have the highest interest in increasing their fertilization. Very intensive farms are served first, beginning with farms having the highest organic nitrogen deficit. Then intensive farms are served, again beginning with the highest organic nitrogen deficit within this intensity level. Depending on the amount of manure given or collected, the available manure per farm is defined.

III.iv.vii Fertilize fields

The available manure is spread onto the respective fields of the farms. Intensive and very intensive farms are also adding mineral fertilizer to their fields to reach an overall nitrogen fertilization level of 200 or 300 kg N/ha, respectively, if the organic manure available does not suffice to meet this demand.

III.iv.viii Harvest fields

Based on the land use type (cropland or grassland field), a given soil fertility factor of each field, the number of hectares cultivated, the number of cuts (if grassland), the amount of nitrogen fertilization, and the average harvest (per cut for grassland, per year for cropland), the feed quantity value (referred to as "net energy content for lactation" in the dairy sector) per field is specified and summed for the entire farm.

III.iv.ix Feed, milk cows, and sell milk

The specified quantity of feed will then be fed to cows. An average productivity of 6748 kg of milk per year and cow is assumed, but the actual productivity depends on the feed quantity value each cow receives. The milk is then sold on the market, whereas extensive farms (intensity scale 1 or 2) receive a higher price per unit milk than intensive farms (intensity scale 3 or 4). This approximates the higher price consumers are often willing to pay for extensively produced products (e.g., organic milk).

III.iv.x Influence ecosystem services
To assess impacts on ecosystem services, indices ranging from 0 (very bad) to 100 (very good) for climate regulation, water quality, habitat quality and soil fertility are calculated. Climate regulation is based on the sum of greenhouse gases emitted by each farm, including CH_4 (determined by the number of cows), N_2O (different emission factors between grassland and cropland, depending on fertilizer input) and CO_2 (grasslands as sink, croplands as source). Soil fertility is enhanced for fields cultivated as grasslands (while depending on the intensity scale of the corresponding dairy farm) and reduced for fields cultivated as croplands summing up to the soil fertility index. As a proxy for the water quality, the overall nitrate output per area is assessed (strength of nitrate output depends on land use and fertilizer intensity). Those grassland fields that are below a certain threshold regarding their number of cuts and their fertilizer input are assumed to have a certain habitat value and by that contribute to the provision of the ecosystem service habitat quality.

III.iv.xi Calculate interim profit
Firstly, field-based income (subsidies) and costs (lease and labor cost) are calculated. Subsidies and lease costs depend on the land use. Labor costs additionally depend on the distance between farms and field and the management intensity (applied fertilizer and – for grassland – number of cuts). In a second step, farms sum up the values on farm level. If the policy option of redistributive subsidy payments is chosen and farms exceed the size limit, a certain share of subsidies is deducted. The input costs on farm level depend on farm size and milk output and, apart from general costs, include the costs for mineral fertilizers used as well as trade costs for manure (both for the donor and the receiver, to account for the needed efforts connected with the manure exchange). The total annual interim profit includes hence on the income side the received subsidies and the received market value and on the expenditure side the land lease, the labor costs as well as the input costs.

III.iv.xii React to policies and set final profit
Policies (if chosen) are implemented in the following order:

- Policy option 1: Determine fines for farms that exceed the allowed level of average organic nitrogen per hectare on their fields after manure trade. The limit of organic nitrogen (100-220 kg/ha), the height of the fine (subsidy reduction of 1-30%) and the percentage of farms that are checked annually (1-100%) have to be set by the observer via the interface
- Policy option 2: Ban grassland conversion if the total share of grasslands falls below the threshold (51-100%) defined by the observer via the interface.
- Policy option 3: Subsidy levels for differing land use and management: Cropland, intensive and extensive grassland are set via the interface (applicable in the next time step). Extensive grasslands are those with no mineral fertilizer application and limited organic nitrogen application (threshold of when an organic nitrogen level is considered extensive is set via the interface to 0-110 kg/ha). If this policy option is switched off in the interface, default values of 300 €/ha subsidies, an organic nitrogen threshold of 110 kg/ha and 350 €/ha additional subsidies for extensive grassland are defined.
- Policy option 4: Redistributive subsidies depending on farm size. The threshold for the farm size (20-100 ha) and the height of the subsidy reduction per ha of farm area exceeding the (5-100%) has to be set by the user. This policy option is implemented in the “calculate interim profit” submodel (see above).

- Policy option 5: Taxes for greenhouse gas and nitrate emissions per dairy farm (height defined by the observer) apply if the area-wide threshold (as defined by the observer) is exceeded.

The final profit then consists of the interim profit minus a potential fine (for exceeding the organic nitrogen limit) minus taxes paid (nitrate and greenhouse gas tax, respectively).

Table 2 Chosen parameter values for the model

procedure	parameter	value	dimension	remarks	source
specify-cow-stuff	amount of manure produced per cow per year	25.4	m³	For a "milk cow": 8000 kg milk/year, 0.9 calves, manure with 7.5% dry mass	(1)
	amount of organic nitrogen content per m³ of manure	4.2	kg/m³	For dairy cattle: Greenland, 7.5% TM. Losses due to storage are considered.	(1)
harvest-fields	Increase in "Net energy content for lactation" depending on N input	0.004	MJ/kg N	The source gives a NEL of 5.69 MJ kg-1 for 70 kg of N fertilization and 6.01 MJ kg-1 for 150 kg. Assuming linear relationship.	(2)
	"Net energy content for lactation" of grass	5.63	MJ/kg dry mass	Mean of all maturity levels of grass for first and second cut (between 5.67 and 7.06 MJ/kg dry mass -> 6.28 MJ/kg dry mass). Normalizing to 0 kg N input: N input recommendation of LfL (Source 2: Table 30/Düngebedarfswert N): 162 kg N/ha/a -> reduction by 162*0.004 (factor above) = 0.65 MJ/kg N	(1), (3)
	Amount of grass harvested per year and cut	3900	kg dry mass/ha	Mean amount of grass harvested per cut in Bavaria in 2018 at different cutting times (between 1700 and 5600 kg dry mass/ha)	(4)
	"Net energy content for lactation" of crop	5.91	MJ/kg dry mass	Mean of all maturity levels of corn (between 6.5 and 6.94 MJ/kg dry mass -> 6.71 MJ/kg dry mass). Normalizing to 0 kg N input: N input recommendation of LfL (Source 2: Futterpflanzen/Silomais (35% TM)/Stickstoffbedarfswert): 200 kg N/ha/a -> reduction by 200*0.004 (factor above, assuming the same) = 0.8 MJ/kg N	(3), (5)
	Amount of crop harvested per year	18414	kg dry mass/ha	Mean amount of corn harvested in Bavaria in 2014 (52610 kg/ha, 35 % dry matter content)	(6)
feed-and-milk-cows	Standard milk output per year and cow	6748	kg	Mean of sold milk per cow of all dairy farm size classes (between 3700 and 7662 kg/cow) adjusted according to corresponding number of farms per class in the "upper-Bavarian grassland belt"	(7)
	Factor for the dependency of milk output per year on the "Net energy content for lactation"	36831	MJ	Food supply recommendation for cows dependent on daily milk production -> demand per cow: 3.3 MJ NEL/kg milk per day (= 1205 MJ NEL/kg milk per year). Based on that linear relation, the factor was calculated for the standard milk output assumed in this model (6748 kg/cow/a).	(3)
influence-ecosystem-services	Yearly CH_4 output per cow	93.7	kg	Mean methane output per cow (MJ/d) of the revised studies for dairy cattle (Table 1 of the study) divided by energy density of methane (55.7 MJ/kg) and multiplied by 365 days	(8)
	Fraction of N_2O emissions relative to total N applied in grasslands	0.0204	-	Mean value of mean emission factors from three grassland sites (single emission factors between 0.003 and 0.058)	(9)
	Fraction of N_2O emissions relative to total N applied in croplands	0.0117	-	Mean value of mean emission factors from four crop types (barley, wheat, potatoes, rape) (single emission factors between 0.002 and 0.047)	(9)

Yearly net CO_2 output per ha of cropland	840	kg	value directly taken	(10)
Yearly net CO_2 storage per ha of grassland	520	kg	value directly taken	(10)
Conversion factor for CH_4	25	-	value directly taken	(11)
Conversion factor for N_2O	298	-	value directly taken	(11)
Factor for nitrate leaching (groundwater / streams) for grassland - depending on nitrogen input	0.09	-	Mean of the minimum (0.03, grassland for cutting) and maximum (0.15, pasture) values of the fraction of nitrate leaching. The values were derived by dividing the leached amount by the total N input.	(12)
Factor for nitrate leaching (groundwater / streams) for cropland - depending on nitrogen input	0.225	-	The study describes leaching losses of 30-60 kg of N losses (mean = 45 kg) with 200 kg N input leading to the respective value of 0.225.	(12)
Factor for soil fertility reduction (= <1), based on changes in C-content if land is used for one year as cropland	0.974	-	Modeled loss of soil carbon stock after 17 years was 36.1% of initial value after conversion of grassland into cropland and then constant. Linear interpolation results in 2.12% loss per year. As the model always only refers to the last year's value of fertility, a mean factor of 0.974 was calculated, leading to the necessary decrease after 17 years.	(13)
Factor for soil fertility increase (= >1), based on changes in C-content if land is used for one year as grassland	1.015, 1.013, 1.010, 1.007 (for intensities 1, 2, 3, 4)	-	Modeled increase in soil carbon stock after 30 years was 57.3% of initial value after conversion of cropland into grassland (Source 13). Linear interpolation results in 1.91% loss per year. As the model always only refers to the last year's value of fertility, a mean factor of 1.015 was calculated, leading to the necessary increase after 30 years. Then, following source 14, the total SOC increase was related to the respective intensity of the farmer, leading to lower increases in soil fertility with higher intensity.	(13), (14)
Definition of high habitat quality: N input threshold / number of cuts per year	0-50 kg/ <= 1 cut	-	inspired by source	(15)
Definition of medium habitat quality: N input threshold / number of cuts per year	50-100 kg/ <= 2 cuts	-	inspired by source	(15)
Definition of low habitat quality: N input threshold / number of cuts per year	>= 100 kg/ > 2 cuts	-	inspired by source	(15)
Factor of consideration for area with medium habitat quality	0.7	-	inspired by source	(15)
Factor of consideration for area with low habitat quality	0.3	-	inspired by source	(15)

calculate-interim-profit	Factor for the calculation of the labor cost for a cropland field, depending on hectare size	633	€/ha	Assuming 2 sowings ("Drillen komplett": 45€/ha), 2 ploughing events (Pflügen komplett, <2 ha Schlaggröße: 115 €/ha), 1 harvest (SF-Silomaishäcksler: 210 €/ha) and 4 fertilizations (25.7€/ha; Allradschlepper 125 PS: 51.4 €/h, assuming 0.5 h/ha) -> 2*45 + 2*115 + 210 + 4*25.7 = 633 €/ha. Values include cost of manpower, machine maintenance and diesel consumption	(16)
	Factor for the calculation of the labor cost for a cropland or grassland field, depending on nitrogen input	0.43	€/kg	Costs of manure fertilization: 1.65 €/m^3. As in procedure "specify-cow-stuff" we assume 3.8 kg N/m^3 manure -> 0.43 €/kg. Not considering manpower and diesel consumption)	(16)
	Factor for the calculation of the labor cost for a cropland field, depending on distance to farm	23.1	€/km	Costs for tractor ride: 51.4 €/h (Allradschlepper 125 PS). Assumed velocity of 40 km/h. Total of 18 rides (9 times there and back) -> 51.4/40*18 = 22.2 €/km. Values include cost of manpower, machine maintenance and diesel consumption	(16)
	Factor for the calculation of the labor cost for a grassland field, depending on hectare size	125.7	€/ha	Assuming 1 fertilization (25.7€/ha; Allradschlepper 125 PS: 51.4 €/h, assuming 0.5 h/ha) and 1 harvest (Mähen komplett: 30 €/ha and Schwadlüfter komplett: 70 €/ha) -> 25.7 + 30 + 70 = 125.7 €/ha. Values include cost of manpower, machine maintenance and diesel consumption	(16)
	Factor for the calculation of the labor cost for a grassland field, depending on distance to farm	5.1	€/km	Costs for tractor ride: 51.4 €/h (Allradschlepper 125 PS). Assumed velocity of 40 km/h. Total of 4 rides (2 times there and back) -> 51.4/40*4 = 5.1 €/km. Values include cost of manpower, machine maintenance and diesel consumption	(16)
	Input costs: per ha price – depending on milk output (<100,000 kg, 100,000-150,000 kg, 150,000-200,000 kg, 200,000-250,000 kg, 250,000-300,000 kg and >300,000 kg)	1025<br>1734<br>1868<br>2163<br>1935<br>2734	€/ha	Input costs of dairy farms in the "upper-Bavarian grassland belt". Input costs include costs for seeds ("Materialaufwand Saat- und Pflanzgut"), pesticides ("Materialaufwand Pflanzenschutz"), animal production ("Materialaufwand Tierproduktion"), electricity, heating, water ("Aufwand Strom, Heizstoffe, Wasser"), and other operational charges	(7)
	Cost factor manure trade (per kg N in manure)	2.4	€/kg	Transport cost: 9 €/m3 manure. As in procedure "specify-cow-stuff" we assume 3.8 kg N/m3 -> 9/3.8= 2.4 €/kg N	(17)
Policy option 3	subsidies-crop-FIT	0-600	€/ha	EU subsidies for Bavaria 2018 per ha: Basic subsidy (Basisprämie, 180 €) + redistribution subsidy (Umwerteilungsprämie, 30-50 € -> mean of 40 €) + young farmers subsidy (Junglandwirteprämie, 44 €) + greening subsidy (Greeningprämie, 86 €) + additional optional subsidies	(18)
	subsidies-grass-int-FIT	0-600	€/ha	EU subsidies for Bavaria 2018 per ha: Basic subsidy (Basisprämie, 180 €) + redistribution subsidy (Umwerteilungsprämie, 30-50 € -> mean of 40 €) + young farmers subsidy (Junglandwirteprämie, 44 €) + greening subsidy (Greeningprämie, 86 €) + additional optional subsidies	(18)

	subsidies-grass-ext-FIT	0-700	€/ha	Subsidies as above + Kulturlandschaftsprogramm (KULAP) subsidy of 55-350 €/ha depending on type of extensive use	(19)
	Norg-ext-threshold	0-110	kg N/ha	In extensive grassland the maximum allowed N fertilizer application rate equals the manure production of 1.4 GVE (Source 1). According to source 2: 1 GVE produces 77 kg N --> 77*1.4 = 110 kg N/ha	(20), (21)
initialize-global-variables	Set the initial market value for the two milk types	0.31/0.48	€/kg	Average values for conventional/organic milk for 2015 in Bavaria - those are set as initial values for milk from intensive/extensive dairy farms respectively	(22)
	Start value of mineral fertilizer price	0.234	€/kg	Mean of ex-stock fertilizer price (Kalkammonsalpeter 27 % N) in December 2018 in Germany	(23)
	Initial values for subsidies (cropland)	300	€/ha	EU subsidies for Bavaria 2018 per ha: Basic subsidy + young farmers subsidy + greening subsidy	(18)
	Initial values for subsidies (grassland intensive)	300	€/ha	EU subsidies for Bavaria 2018 per ha: Basic subsidy + young farmers subsidy + greening subsidy	(18)
	Initial values for subsidies (grassland extensive)	350	€/ha	EU subsidies for Bavaria 2018 per ha: Basic subsidy + KULAP subsidy for extensive grassland use without the use of mineral fertilizer (see KULAP: Extensive Grünlandnutzung für Raufutterfresser mit Verzicht auf Mineraldüngung: B20 (max. 1.40 GV/ha)	(18), (19)
	Leasing price per hectare of cropland	396	€/ha	Average leasing price for Bavaria in 2016	(24)
	Leasing price per hectare of grassland	221	€/ha	Average leasing price for Bavaria in 2016	(24)

References

(1) https://www.lfl.bayern.de/mam/cms07/publikationen/daten/informationen/leitfaden-duengung-acker-gruenland_gelbes-heft_lfl-information.pdf

(2) Angeringer, W., Starz, W., & Karrer, G. (2013). Wirkung verschiedener Nutzungsintensitäten auf montane Goldhaferwiesen im Biolandbau.

(3) Bayerisches Landesamt für Landwirtschaft (2017). Gruber Tabelle zur Fütterung der Milchkühe, Zuchtrinder, Schafe, Ziegen. 43. Aufl. Freising: LfL-Information.

(4) https://www.lfl.bayern.de/mam/cms07/ite/dateien/voralpineshügelland.pdf

(5) https://www.lfl.bayern.de/mam/cms07/iab/dateien/basisdaten_20180730_9a.pdf

(6) https://www.statistik.bayern.de/veroeffentlichungen/epaper.php?pid=42311&t=1

(7) https://www.stmelf.bayern.de/bfm/index.do

(8) Ellis, J. L., Kebreab, E., Odongo, N. E., McBride, B. W., Okine, E. K., & France, J. (2007). Prediction of methane production from dairy and beef cattle. Journal of dairy science, 90(7), 3456-3466.

(9) Dobbie, K. E., McTaggart, I. P., & Smith, K. A. (1999). Nitrous oxide emissions from intensive agricultural systems: variations between crops and seasons, key driving variables, and mean emission factors. Journal of Geophysical Research: Atmospheres, 104(D21), 26891-26899.

(10) Vleeshouwers, L. M., & Verhagen, A. (2002). Carbon emission and sequestration by agricultural land use: a model study for Europe. Global change biology, 8(6), 519-530.

(11) Solomon, S., Qin, D., Manning, M., Averyt, K., & Marquis, M. (Eds.). (2007). Climate change 2007-the physical science basis: Working group I contribution to the fourth assessment report of the IPCC (Vol. 4). Cambridge university press.

(12) Di, H. J., & Cameron, K. C. (2002). Nitrate leaching in temperate agroecosystems: sources, factors and mitigating strategies. Nutrient cycling in agroecosystems, 64(3), 237-256.

(13) Poeplau, C., Don, A., Vesterdal, L., Leifeld, J., Van Wesemael, B. A. S., Schumacher, J., & Gensior, A. (2011). Temporal dynamics of soil organic carbon after land-use change in the temperate zone–carbon response functions as a model approach. Global change biology, 17(7), 2415-2427.

(14) Khalil, I. M. (2019). Startegic Management of Grazing Systems to Maintain and Increase Organic Carbon in Soils [Online First]. IntechOpen. DOI: 10.5772/intechopen.84341.

(15) Boch, S., Allan, E., Birkhofer, K., Bossdorf, O., Blüthgen, N., Christ-Breulmann, S., ... & Hemp, A. (2016). Extensive und jährlich wechselnde Nutzungsintensität fördert den Artenreichtum im Grünland. Anliegen Natur, 38(1), 86-95.

(16) Verrechnungssätze 2018, Maschinenring Aibling, https://www.mr-aibling.de/verrechnungssaetze.html

(17) https://www.lksh.de/fileadmin/dokumente/Bauernblatt/PDF_Toepper_2014/BB_11_15.03/35-38_Reckleben.pdf;

(18) http://www.stmelf.bayern.de/agrarpolitik/foerderung/000958/index.php

(19) https://www.stmelf.bayern.de/mam/cms01/agrarpolitik/dateien/massnahmenuebersicht_kulap.pdf

(20) https://www.landwirtschaftskammer.de/foerderung/laendlicherraum/aum/gruenland.htm

(21) https://www.lwk-niedersachsen.de/download.cfm/file/12989.html

(22) https://www.lfl.bayern.de/mam/cms07/publikationen/daten/informationen/statistik-bayerische-milchwirtschaft-2015_lfl-information.pdf (Table 16)

(23) https://markt.agrarheute.com/duengemittel-4/stickstoffduenger-20

(24) https://www.statistik.bayern.de/presse/archiv/142_2017.php
